# Novel test method to measure time-cure superposition shift factors of filled-thermoset under *isocure* testing conditions

**DOI:** 10.1007/s10853-026-12287-w

**Published:** 2026-02-03

**Authors:** Sukrut Prashant Phansalkar, Bongtae Han

**Affiliations:** Department of Mechanical Engineering, University of Maryland, College Park, MD USA

## Abstract

Despite the solid theoretical foundation of time-cure superposition, the time-cure superposition (TCS) shift factors reported in the literature do not support the theory very well. The discrepancy stems from the *non*-*isocure* test conditions used in the tests. This study proposes a novel method to eliminate the inherent problems of existing techniques to measure the TCS shift factors, i.e., to measure them under *isocure* test conditions. The proposed method optimizes a test procedure while offering sufficient relaxation but producing no or negligible additional curing during testing. Optimization requires a complete understanding of curing behavior not only in the chemically-controlled domain but also in the diffusion-controlled domain. The method is implemented for an epoxy-based molding compound. Portions of the storage master curves are obtained at four partially-cured states $(p = 0.6, 0.7, 0.8, 0.9)$, and they are normalized by the corresponding equilibrium modulus. The normalized curves are subsequently shifted to determine the TCS shift factors using the master curve of fully-cured specimen as a reference. The results show excellent overlaps over the entire curing range after shifting, corroborating that the proposed method is accurate and effective. Validity of the time-cure superposition and applicability of the time–temperature superposition to partially-cured specimens are also confirmed using the test data used to determine the TCS shift factors as well as additional test data.

## Introduction

There are numerous high-temperature manufacturing processes using thermosets. Notably, semiconductor packaging manufacturing processes utilize thermosets, filled with inorganic fillers (e.g., silica particles), to encapsulate and protect semiconductor chips [1–3]. The processes typically involve curing the filled-thermosets under high-temperature and high-pressure conditions. Thus, the viscoelastic properties of the filled-thermosets as a function cure-extent (hereinafter, referred as “cure-dependent” for conciseness) are critically required to predict the residual stresses after the molding process.

Despite high filler content (as high as 90% wt./wt.), the molding compound exhibits significant time-dependent behavior [4–6], which produces large residual stress after molding [7–9]. Residual stresses often cause various manufacturing and reliability challenges, most significantly excessive warpage [10–13]. It is, thus, important to predict the behavior of molded packages during and after molding for process optimization, which obviously cannot be done without cure-dependent viscoelastic properties.

The theoretical foundation of cure-dependent viscoelastic behavior was established by Adolf and Martin [14]. In their original studies, the fundamental description of the complex viscoelastic evolution of an unfilled thermoset during curing was provided, which became the basis of the concept, known as the time-cure superposition (TCS).

The TCS concept was significantly advanced by a research group of Delft University of Technology, led by Jansen and Ernst [15–20]. They clearly identified two critical cure-dependent properties to be measured for prediction of the viscoelastic behavior during curing: they are (1) the cure-dependent equilibrium modulus, ${E}_{\infty }(p)$, and (2) the time-cure superposition (TCS) shift factors, ${a}_{p}(p)$. Despite this important advancement, the measured properties reported in Refs. [15–20] did not reflect the theory very well.

Recently, the problems associated with measurements of the cure-dependent equilibrium modulus were resolved completely by a novel test method proposed in Ref. [21]. In the method, the test temperature was set to be much higher than the glass transition temperature of a partially-cured specimen, ${T}_{g}(p)$, so that complete relaxation occurred instantaneously at the test temperature. A very short duration (≤ 10 s) monotonic testing was conducted to determine the cure-dependent equilibrium modulus. This short duration testing time eliminated the undesired additional curing during testing, resulting in accurate measurement of the cure-dependent equilibrium modulus.

Unlike the equilibrium modulus, a test data for measurement of TCS shift factors, ${a}_{p}(p)$, must be obtained over many decades because a significant portion of the master curve should be used for accurate shifting. In Refs. [15–20, 22, 23], the routinely-practiced multi-frequency temperature (MFT) sweeping method, a dynamic mechanical analyzer (DMA), was used to obtain the Young’s modulus master curves of several partially-cured epoxy-based molding compound (EMC) specimens. Although optimized, the MFT sweeping required over an hour of testing time near or above the glass transition region. Undesired additional curing of partially-cured specimens was unavoidable, which caused significant uncertainties in the master curves. This effect is rather clear in the reported data. The normalized master curves obtained from partially-cured specimens did not overlap well, which was contrary to the behavior predicted by the theory.

In this study, a novel method to measure ${a}_{p}(p)$ is proposed to eliminate the inherent problems of the existing techniques, i.e., measurements of ${a}_{p}(p)$ under *isocure* test conditions. The goal is achieved by optimizing a test procedure while offering sufficient relaxation but producing no or negligible additional curing.

The theoretical background of time–temperature superposition (TTS) and time-cure superposition (TCS) is reviewed briefly first. The detailed description and implementation of the proposed method using an EMC are followed. Finally, the validity of TCS and the applicability of TTS to partially-cured specimens are discussed using the test data used to determine ${a}_{p}(p)$ as well as additional test data.

## Theoretical background

### Temperature-dependent viscoelasticity and time–temperature superposition (TTS)

For polymers exhibiting the thermo-rheologically simple behavior, the viscoelastic behavior under a stress relaxation testing condition can be expressed by the generalized Maxwell model using the time–temperature superposition (TTS) shift factors, ${a}_{T}(T)$, as [24]:

1 $$E\left( {t,T} \right) = E_{\infty } + \left( {E_{G} - E_{\infty } } \right)\sum\limits_{{i = 1}}^{N} {e_{i} \exp \left( { - \frac{t}{{a_{T} \left( T \right)\tau _{i} }}} \right)} ;\;e_{i} = \frac{{E_{i} }}{{E_{G} - E_{\infty } }}$$

where ${e}_{i}$ and ${\tau }_{i}$ are the normalized Maxwell model constants of Young’s modulus (also known as the relaxation ratios in commercial FEA packages) and the relaxation times, respectively; ${E}_{G}$ and ${E}_{\infty }$ are the Young’s glassy and equilibrium modulus, respectively, and ${a}_{T}\left(T\right)$ is the shift factor which provides a relationship between the relaxation times as ${\tau }_{i}\left(T\right)={a}_{T}\left(T\right){\tau }_{i}$.

**Figure 1:**
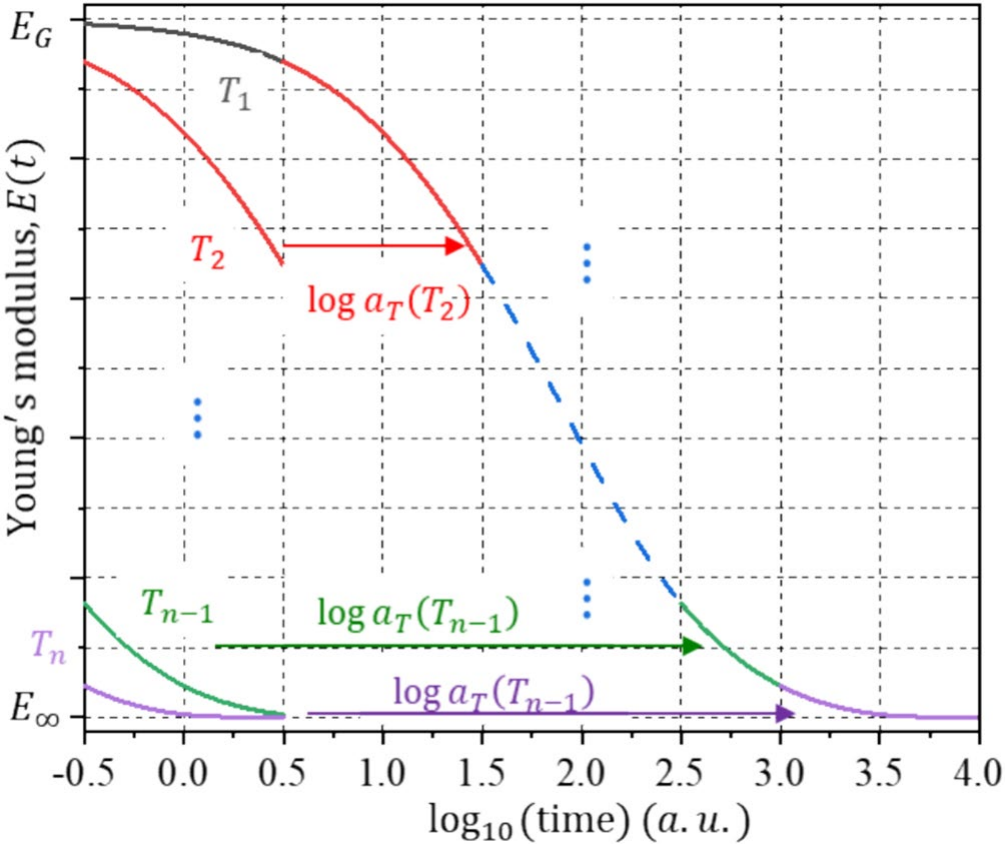
Schematic illustration of TTS shifting to form the master curve.

In practice, several relaxation tests are conducted at various temperatures to produce short segments of the master curve. These short segments are shifted along the log time axis to create a complete relaxation master curve [24, 25]. This is well-known as time–temperature superposition (TTS). This is illustrated schematically in Fig. 1. Several segments at temperatures, ${T}_{1}, {T}_{2},\dots , {T}_{n}$ obtained from a stress relaxation test are shown. The segments at ${T}_{2, }{T}_{3},\dots ,{T}_{n}$ are shifted along the log time scale until they overlap with ${T}_{1}$ (also known as the reference temperature, ${T}_{\mathrm{ref}}$) to create one continuous master curve. The amount of shifting is the TTS shift factor, ${a}_{T}(T)$.

### Cure-dependent viscoelasticity and time-cure superposition (TCS)

For a polymer at a cure-extent, $p$, the relaxation modulus, can be expressed using the Prony series as:

2 $$E\left( {t,p} \right) = E_{\infty } \left( p \right) + \left[ {E_{G} - E_{\infty } \left( p \right)} \right]\sum\limits_{{i = 1}}^{N} {e_{i} } \left( p \right)\exp \left( { - \frac{t}{{\tau _{i} \left( p \right)}}} \right);\;e_{i} \left( p \right) = \frac{{E_{i} \left( p \right)}}{{E_{G} - E_{\infty } \left( p \right)}}$$

where ${E}_{\infty }(p)$ is the cure-dependent equilibrium modulus; and ${e}_{i}(p)$ and ${\tau }_{i}(p$) are the cure-dependent normalized Maxwell model constants and the relaxation times, respectively.

It has been known that clusters formed during gelation remain geometrically self-similar during a curing process; i.e., the shapes of clusters do not change after the gel point, but only the size becomes larger while $p$ increases [14]. This implies that cure-extent does not alter the relaxation mechanism but affects only the rate of relaxation behavior. Therefore, the increase in cluster sizes can be described as the same material with a proportionally longer relaxation time (larger clusters relax more slowly) and a larger equilibrium modulus [26]. This behavior is known as the time-cure superposition (TCS) [14, 26].

The TCS concept implies that the normalized Maxwell model constants are no longer cure-dependent, i.e., ${e}_{i}(p)\approx {e}_{i}$. Similar to the shift factors in the time–temperature superposition (TTS), the time-cure superposition (TCS) shift factors, ${a}_{p}\left(p\right)$, provides a relationship between the relaxation times as ${\tau }_{i}\left(p\right)={a}_{p}\left(p\right){\tau }_{i}$. Then, the cure-dependent viscoelastic behavior under TCS can be described as:

3 $$E\left( {t,p} \right) = E_{\infty } \left( p \right) + \left[ {E_{G} - E_{\infty } \left( p \right)} \right]\mathop \sum \limits_{i = 1}^{N} e_{i} \exp \left( { - \frac{t}{{a_{p} \left( p \right)\tau_{i} }}} \right)$$

Theoretically, the TCS shift factors, ${a}_{p}\left(p\right)$, can be obtained from the master curves measured at various cure-extents. First, Eq. 3 can be normalized as [20]:

4 $$\overline{E}\left( {t,p} \right) = \frac{{E\left( {t,p} \right) - E_{\infty } \left( p \right)}}{{E_{G} - E_{\infty } \left( p \right)}}$$

The normalized master curves can be shifted horizontally on the log time scale to determine the shift factors.

**Figure 2:**
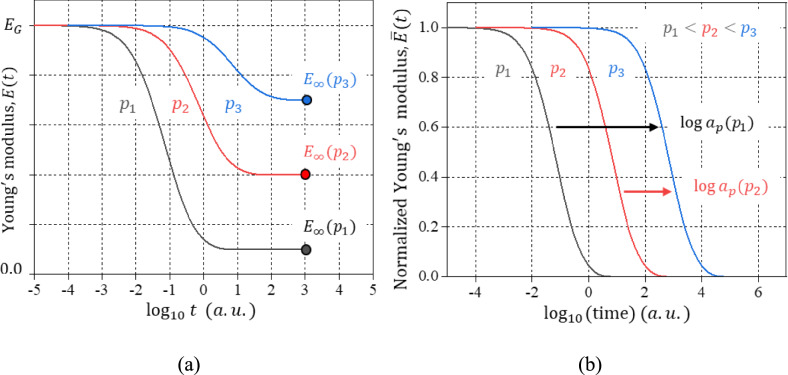
Schematic illustration to determine the time-cure superposition shift factors: **a** the master curves at three different cure-extents $({p}_{1}<{p}_{2}<{p}_{3})$ are shown and **b** the curves rescaled by Eq. 4 are shifted horizontally on the log time scale to determine the shift factors.

This is illustrated in Fig. 2. In (a), the master curves at three different cure-extents $({p}_{1}<{p}_{2}<{p}_{3})$ are shown. As the cure-extent increases, the polymer relaxes more slowly and the equilibrium modulus, ${E}_{\infty }(p)$, increases. The corresponding curves after normalized by Eq. 4 are shown in (b). They shifted along the log time axis to determine the TCS shift factors.

## Proposed method

For measurement of the time-cure superposition (TCS) shift factor, ${a}_{p}(p)$, a significant portion of time-dependent behavior (known as the $\alpha -$ relaxation region) should be used for accurate shifting. Even for half the portion of the master curve for shifting, test data must be obtained over many decades of time.

The cumulative test duration, ${t}_{m}$, to obtain the portion of the master curve is governed by the number of temperature steps, ${T}_{1},{T}_{2},..{T}_{n}$ where ${T}_{n}$ is the highest test temperature which will be referred to as ${T}_{\mathrm{max}}$. Each temperature step takes the test ${i}^{\mathrm{th}}$ test duration, ${t}_{\mathrm{test}}^{i},$ and the heating time between each step, ${t}_{\mathrm{ramp}}^{i+1}$. This can be expressed simply by:

5 $$t_{m} = \mathop \sum \limits_{i = 1}^{N} t_{{{\mathrm{test}}}}^{i} + \mathop \sum \limits_{i = 1}^{N} t_{{{\mathrm{ramp}}}}^{i + 1}$$

**Figure 3:**
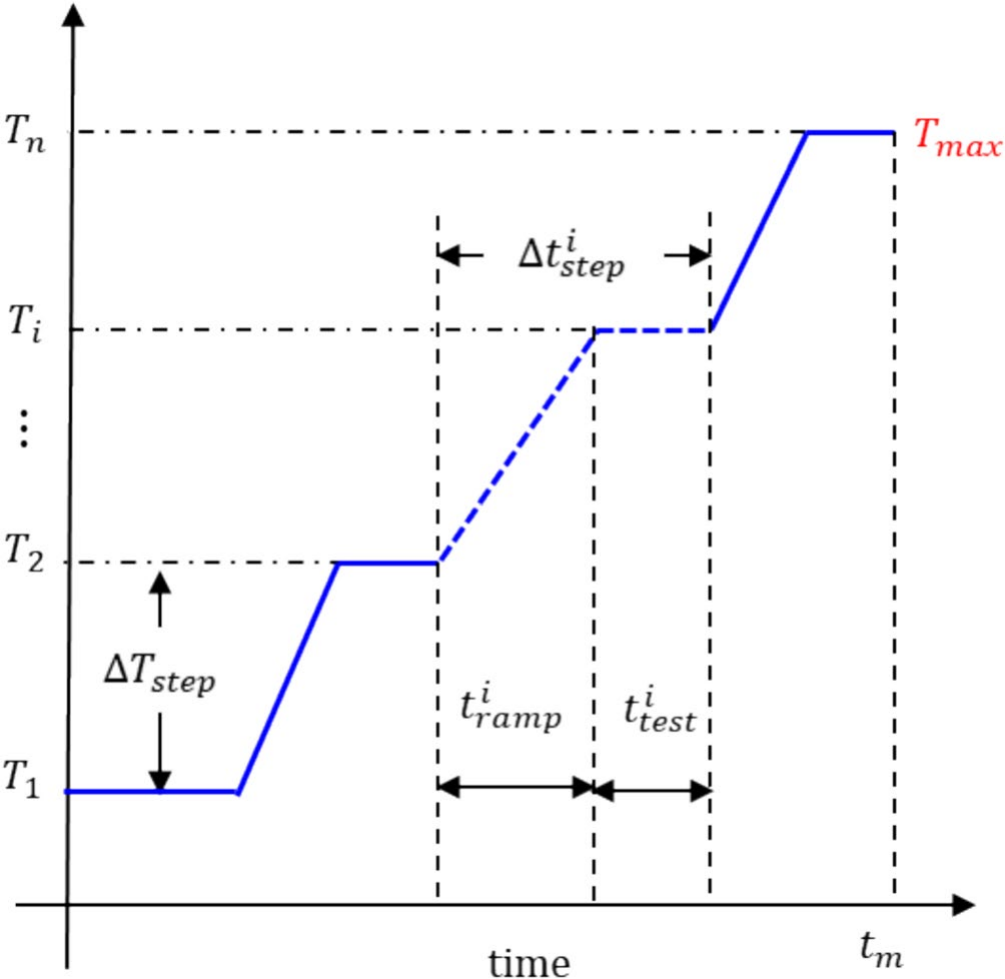
Illustration of temperature steps and the cumulative measurement durations.

The number of temperature steps and the cumulative measurement durations are illustrated schematically in Fig. 3.

It is tempting to consider the approach proposed for the equilibrium modulus measurement used in Ref. [21] to shorten the testing time, i.e., conducting tests at a test temperature, ${T}_{\mathrm{test}}$, much higher than the cure-dependent glass transition temperature of partially-cured specimen, ${T}_{g}(p)$. This approach cannot offer an *isocure* state simply because the test duration is much longer for the shifting factor measurement. Therefore, the values of ${T}_{\mathrm{max}}$ that are lower or near ${T}_{g}(p$) should be considered instead.

**Figure 4:**
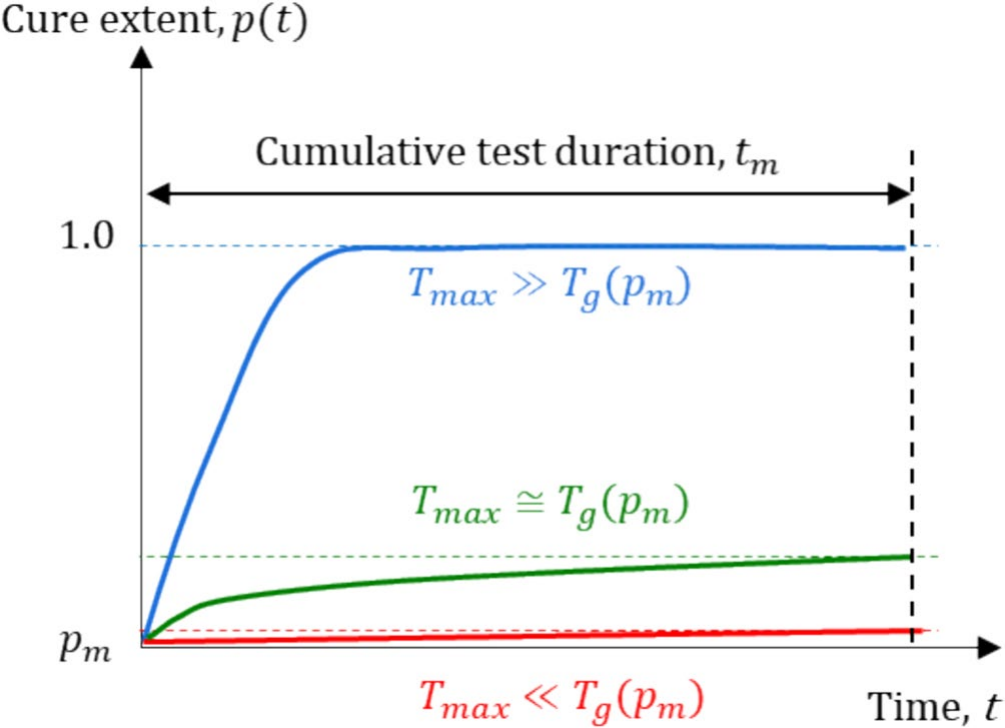
Illustration of additional curing of partially-cured specimen at cure extent of ${p}_{m}$ under different test temperatures.

These conditions are illustrated schematically in Fig. 4, where the additional curing during testing is shown for three different cases. When ${T}_{\mathrm{max}}\gg {T}_{g}(p)$, the reaction kinetics is governed by the chemical control, and high curing rates result in significant additional curing during testing (blue line). When ${T}_{\mathrm{max}}\ll {T}_{g}(p)$, however, the reaction kinetics is completely dominated by the diffusion control, i.e., the curing rate becomes virtually zero (red line). The additional curing is negligible during testing, but testing duration, ${t}_{m}$, to achieve a $\alpha -$ relaxation region becomes too long to be practical [27].

The ideal condition is illustrated as a green-line, where ${T}_{\mathrm{max}}$ is carefully chosen to allow relaxation while offering nearly isocure state. In other words, at the proper ${T}_{\mathrm{max}}$, the reaction rate is lowered significantly by the contribution of the diffusion control, but relaxation is still sufficiently fast to produce the required portion of the master curve.

A proper selection of ${T}_{\mathrm{max}}$ is a critical task for the TCS shift factor measurement, which requires complete understanding of curing behavior under the diffusion-controlled domain. The reaction kinetics that accounts for the effects of diffusion control can be expressed as [28]:

6 $${\left(\frac{dp}{dt}\right)}_{\mathrm{all}} = [1 - \zeta \left( {T,p)} \right]{\left(\frac{dp}{dt}\right)}_{\mathrm{ch}}$$

$\mathrm{where} {\left(\frac{dp}{dt}\right)}_{\mathrm{all}}$ is the overall reaction rate; ${\left(\frac{dp}{dt}\right)}_{\mathrm{ch}}$ is the chemically-controlled reaction rate; and $\zeta$ is a cure-extent and cure-temperature dependent function that accounts for the diffusion control to the cure kinetics. The value of $\zeta$ evolves from 0 to 1; the value becomes “zero” when $T\gg {T}_{g}(p)$, while it becomes “unity” when $T\ll {T}_{g}(p)$.

Many functions have been proposed to describe the diffusion-controlled behavior, but there has been no single function that has been widely used in the community. In this study, the logistic function is selected to describe the diffusion control behavior, which is written as:

7 $$\zeta \left( {\tilde{T}} \right) = 1 - \frac{1}{{1 + \exp \left( { - A\left( {\tilde{T} - B} \right)} \right)}}\;{\mathrm{where}}\;\tilde{T} = T - T_{g} \left( p \right)$$

where $A$ and $B$ are material constants. The values $\zeta$ around ${T}_{g}(p)$ must be determined experimentally to be able to properly select ${T}_{\mathrm{max}}$ for TCS shift factor measurements. The proposed method to measure the TCS shift factors can be summarized as:

1. Measure the cure kinetics under the diffusion-controlled domain, i.e., $\zeta \left(T\right)$ below ${T}_{{g}_{\infty }}$, which is the glass transition temperature of fully-cured EMC.
2. Determine the number of temperature steps, the highest test temperature, ${T}_{\mathrm{max}},$ and the cumulative measurement duration, ${t}_{m}$, for isocure conditions.
3. Fabricate partially-cured specimens.
4. Obtain half the master curve of each partially-cured specimens using TTS.
5. Determine the TCS shift factors from the normalized master curves.

## Implementation

The curing behavior under the diffusion control domain is characterized first. The result is utilized to ensure the required *isocure* state for measurement of the cure-dependent shift factors.

### Curing behavior

A commercially available EMC was used in the study. The base polymer of the EMC is an epoxy resin with a phenolic hardener, and it contains a large amount of fused-silica filler (87% wt./wt.). It has been reported that the rate constant of EMC is independent (or a very weak function) of the cure-extent, and thus, an autocatalytic model known as the Kamal-Sourour (K-S) model describes the cure kinetics of EMC in the chemically-controlled domain faithfully [29, 30]. The model is expressed as:

8 $$\frac{{{\mathrm{d}}p}}{{{\mathrm{d}}t}} = k_{0} \exp \left( { - \frac{{E_{a} }}{{{\mathrm{RT}}}}} \right)\left( {1 - p} \right)^{n} \cdot p^{m}$$

where $dp/dt$ is a curing rate; $p$ is a cure-extent; $T$ is the curing temperature (in Kelvin); ${k}_{0}$ and ${E}_{a}$ are a pre-exponential factor and an activation energy, respectively; and $R$ is the ideal gas constant (8.314 $\text{J mo}{\mathrm{l}}^{-1}{\mathrm{K}}^{-1}$).

The model constants obtained from previous analysis [29] are:

9 $$\left\{ {\begin{array}{*{20}c} {\ln A \left( {{\mathrm{s}}^{ - 1} } \right)} \\ {E_{a} \left( {{\mathrm{kJ}}/{\mathrm{mol}}} \right)} \\ n \\ m \\ \end{array} } \right\} = \left\{ {\begin{array}{*{20}c} {17.56} \\ {75.54} \\ {1.12} \\ {0.63} \\ \end{array} } \right\}$$

The cure-dependent glass transition temperature, ${T}_{g}(p)$, of the material was measured in the previous study. The values of ${T}_{g}(p)$ were described faithfully by the DiBenedetto (DB) equation [31] as:

10 $$T_{g} \left( p \right) = T_{g0} + (T_{g\infty } - T_{g0} )\frac{\lambda p}{{1 - \left( {1 - \lambda } \right)p}}$$

where ${T}_{g0}$($=23.85^\circ \mathrm{C}$) and ${T}_{g\infty } (=130.73^\circ \mathrm{C})$ are the glass transition temperatures of uncured and fully-cured EMC, respectively; and the material parameter, $\lambda$, is 0.378 [21].

**Figure 5:**
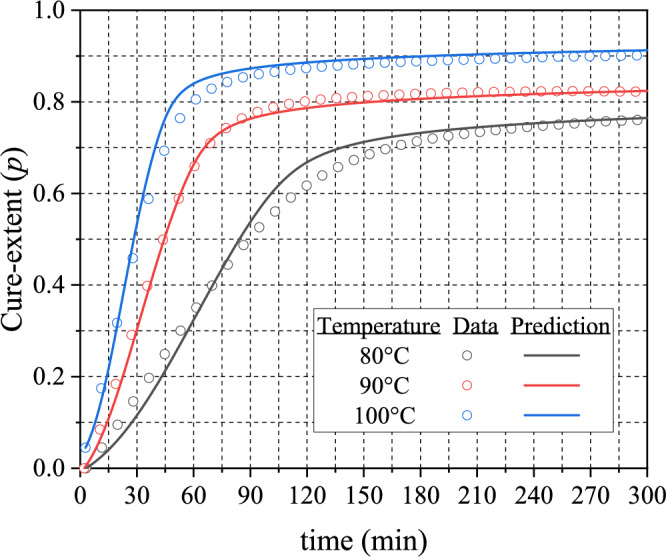
Results of isothermal testing conducted at ${T}_{\mathrm{test}}$ below ${T}_{g\infty }$; the lines represent the prediction by Eq. 6.

Several additional isothermal DSC tests were conducted at curing temperatures below ${T}_{g\infty }$ to document the diffusion-controlled behavior of EMC. Cure-extents obtained from the tests are shown in Fig. 5 (scatter plots). The material constants of Eq. 7 were determined from non-linear regression: $A=0.213^\circ {\mathrm{C}}^{-1}$ and $B=7.723^\circ \mathrm{C},$ respectively. The model prediction is also shown in Fig. 5. It is evident that the model predicts faithfully the change in cure-extent over time at measurement temperatures below ${T}_{g\infty }$.

### MFT sweeping tests of fully and partially-cured specimens

**Figure 6:**
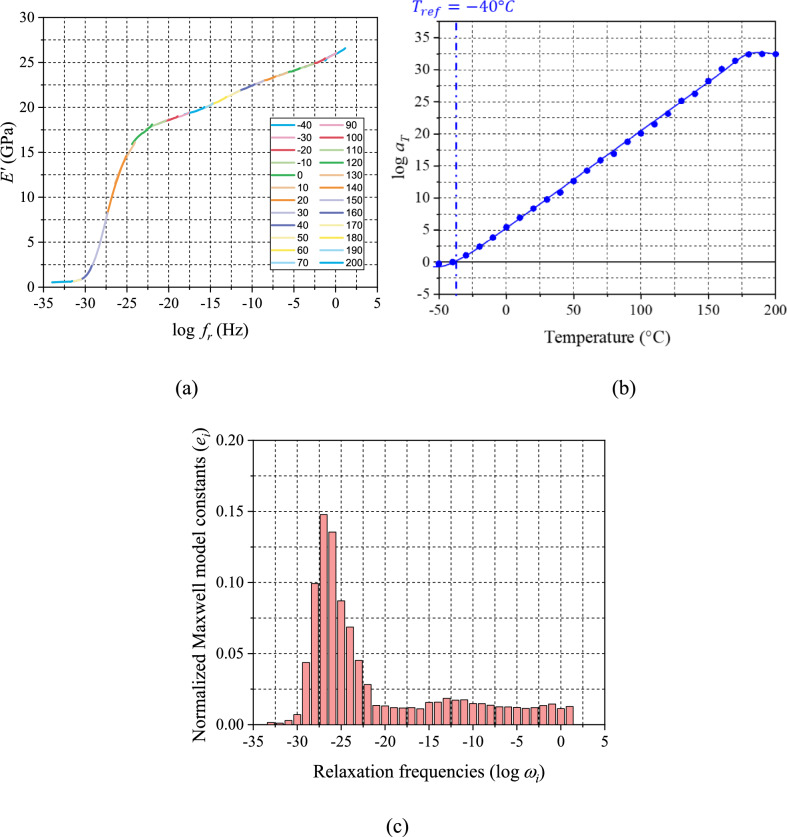
Results of DMA testing: **a** Storage modulus master curve (${T}_{\mathrm{ref}}=-40^\circ \mathrm{C}$), **b** TTS shift factors, and **c** the normalized Maxwell constants as a function of relaxation frequency.

A fully-cured specimen of 50 mm × 10 mm × 3 mm was fabricated by a custom-designed mold [31]. EMC was cured at 175 °C for 2 h to ensure complete cure. An advanced MFT procedure developed in Ref. [25] was employed to measure the storage modulus over (1) a temperature range of − 40 to 200 °C (10 °C/step) and (2) a frequency range of 0.1–25 Hz (5 points per decade) using the three-point bending fixture of a commercial DMA machine (TA DMA850). The TTS shifting was followed to generate a master curve and a set of shift factors. The storage modulus master curve and the corresponding shift factors are shown in Fig. 6a and b, respectively, where the reference temperature, ${T}_{\mathrm{ref}}$, was $-\hspace{0.17em}40^\circ \mathrm{C}$, which corresponds to ${T}_{g0 }$.

The Prony series was determined from the storage modulus master curve, which are expressed as:

11 $$E^{\prime}\left( \omega \right) = E_{\infty } + \left( {E_{G} - E_{\infty } } \right)\mathop \sum \limits_{i = 1}^{N} e_{i} \frac{{\omega^{2} }}{{\omega_{{{\mathrm{rel}},i}}^{2} + \omega^{2} }}$$

where ${\omega }_{\mathrm{rel},i} (\equiv 1/{\tau }_{i})$ is the relaxation frequency. The constants can be used to define the master curve in the time domain using Eq. 1. The normalized Maxwell model constants and the corresponding relaxation frequency obtained from (a) are shown in (c).

The partially-cured specimens (50 mm × 10 mm × 3 mm) at three cure-extents ($p\hspace{0.17em}=\hspace{0.17em}$0.6, 0.7, 0.8 and 0.9) were also fabricated by the same mold. The curing schedules used to fabricate the specimens, based on the K-S model, are shown in Table 1.

**Table 1:** Curing temperature and time used to fabricate partially-cured specimens

Cure-extent ($p$)	Curing temperature (°C)	Curing time (mins)
0.6	120	5.8
0.7	125	6
0.8	130	5
0.9	140	4

The DMA machine offered the lowest temperature of ${T}_{1}=-\hspace{0.17em}40^\circ \mathrm{C}$. The same parameters used for the fully-cured specimen were used the partially-cured specimens: $\Delta {T}_{\mathrm{step}}=10^\circ \mathrm{C}/\mathrm{step}$ and a frequency range of 0.1–25 Hz. The ramp time, ${t}_{\mathrm{ramp}},$ between each temperature was 10 min, and the frequency sweeping duration, ${t}_{\mathrm{test}}$, was 75 s.

The cure-extent continues to increase during testing. A percentage increase of cure-extent during testing, $\eta \left(\%\right)$, is defined as:

12 $$\eta \left( \% \right) = \frac{{{\Delta }p\left( t \right)}}{{p_{m} }} \times 100$$

where $\Delta p\left(t\right)$ is an additional curing amount during testing; and ${p}_{m}$ is the cure-extent of a partially-cure specimen before testing. The value of ${p}_{m}$ can be readily estimated by using the cure kinetics model. To ensure an *isocure* state, $\eta \left(\%\right)$ should not exceed a certain critical value, ${\eta }_{c}$. The critical value of ${\eta }_{c}$ is assumed be 1% in this study.

**Figure 7:**
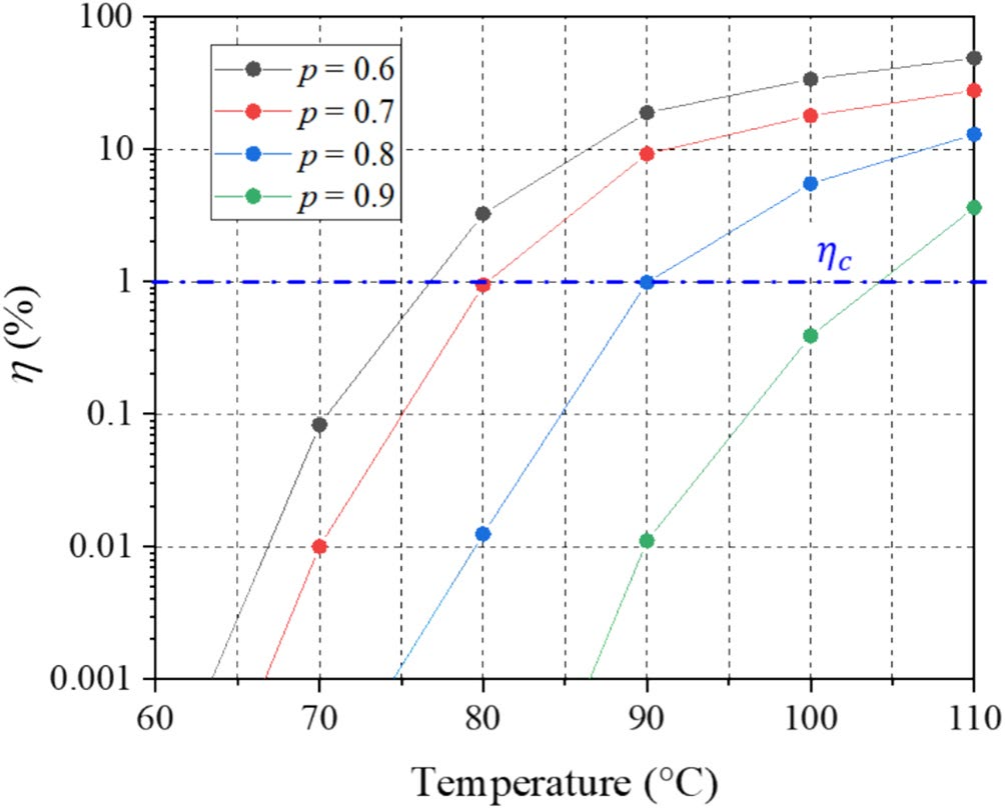
Estimation of cure-extent change during MFT testing.

This temperature profile (see Fig. 3) was used to calculate $\Delta p(t)$ at the end of each $\Delta {T}_{\mathrm{step}}$ using Eqs. 6 and 7. Then, the percentage change in cure-extent, $\eta \left(\%\right)$, was estimated using Eq. 7. Figure 7 shows $\eta (\%)$ for $p=0.6, 0.7, 0.8,\text{ and }0.9$ as a function of temperature, where the data points show the change at the end of each temperature step considering $\Delta {T}_{\mathrm{step}}=10^\circ \mathrm{C}/\mathrm{step}$. The critical value, ${\eta }_{c}=1\%$, is marked using a blue line. Any temperature below the line can be used as ${T}_{\mathrm{max}}$.

Considering higher relaxation behavior at low cure-extents ($p=0.6 \text{ and } 0.7$), the actual values for ${T}_{\mathrm{max}}$ used in the testing were 50, 70, 90, and 100 °C for $p=0.6, 0.7, 0.8,\text{ and }0.9$, respectively.

**Figure 8:**
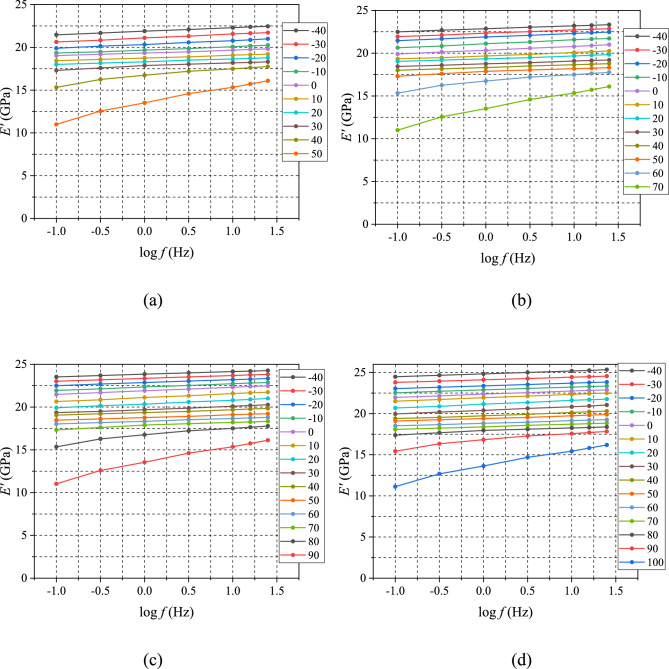
Storage modulus data of partially-cured specimens obtained by MFT sweeping tests: **a** $p=0.6$, **b** $p=0.7$, **c** $p=0.8,$ and **d** $p=0.9$.

**Figure 9:**
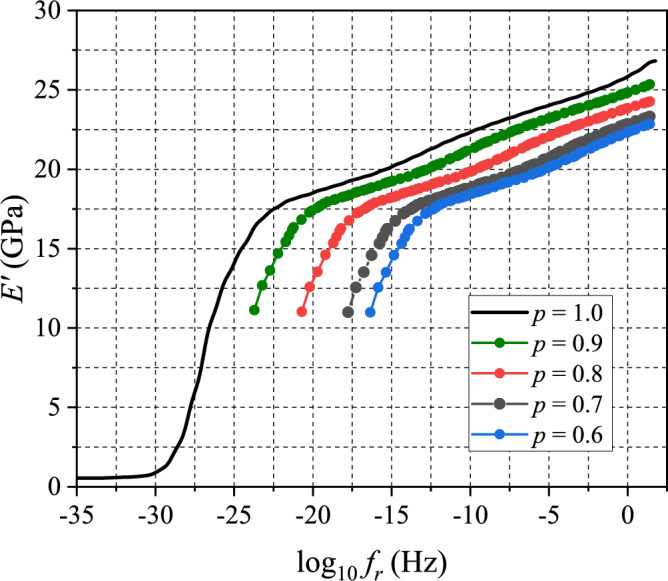
Storage modulus master curves of partially-cured specimens as well as a full-cured specimen obtained from MFT sweeping test, where the reference temperature is ${T}_{\mathrm{ref}}=-40^\circ \mathrm{C}$.

The test results of the partially-cured specimens under the above conditions are shown in Fig. 8. The storage modulus data were shifted to create sections of master curve at ${T}_{\mathrm{ref}}= -40^\circ \mathrm{C}$. The sections of master curves obtained from the data are shown in Fig. 9 together with the master curve of the fully-cured EMC (Fig. 6a).

### Calculations of time-cure superposition shift factors

As stated earlier, the master curves of partially-cured specimens must be normalized by the cure-dependent equilibrium modulus, ${E}_{\infty }(p)$, to determine the TCS shift factors, ${a}_{p}(p)$, before shifting. A percolation theory-based evolution model was used to describe the cure-dependent Young’s equilibrium modulus in Ref. [21]. The equilibrium modulus evolution was expressed by the following equation:

13 $$E_{\infty } \left( p \right) = E_{\infty }^{f} \left( {\frac{{p^{2} - p_{{{\mathrm{gel}}}}^{{2}} }}{{1 - p_{{{\mathrm{gel}}}}^{{2}} }}} \right)^{n}$$

where ${E}_{\infty }^{f}$ ($=$ 534.5 MPa) is the equilibrium modulus of a fully-cured polymer, ${p}_{\mathrm{gel}}$ is the gel point and $n$ is the evolution exponent. The same EMC was tested in this study, and the constants determined in Ref. [21] were employed for the analysis: ${E}_{\infty }^{f}$ $=$ 534.5 MPa, ${p}_{\mathrm{gel}}=0.39$ and $n=3.41$. More details about the percolation model constants can be found in Ref. [21].

**Figure 10:**
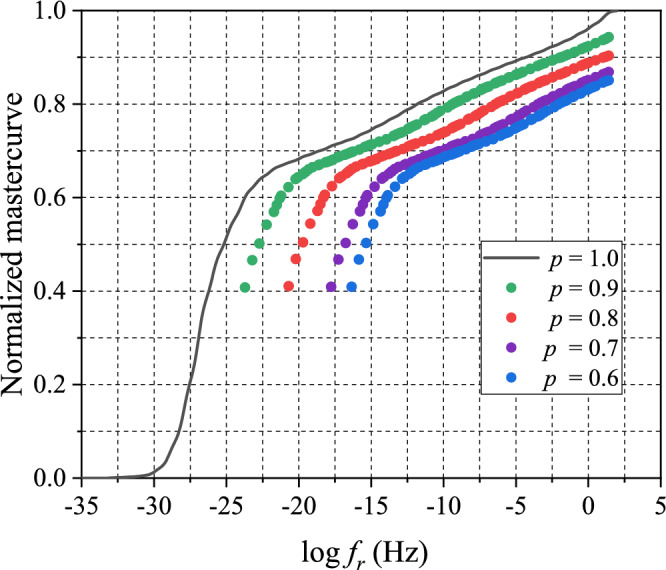
Normalized storage master curves for partially-cured EMC at ${T}_{\mathrm{ref}}=-40^\circ \mathrm{C}$.

The storage modulus master curves (Fig. 9) were normalized by ${E}_{\infty }(p)$. The normalized master curves of partially-cured specimens at various cure extents including that of the fully-cured specimen ($p=1$) are shown in Fig. 10.

**Figure 11:**
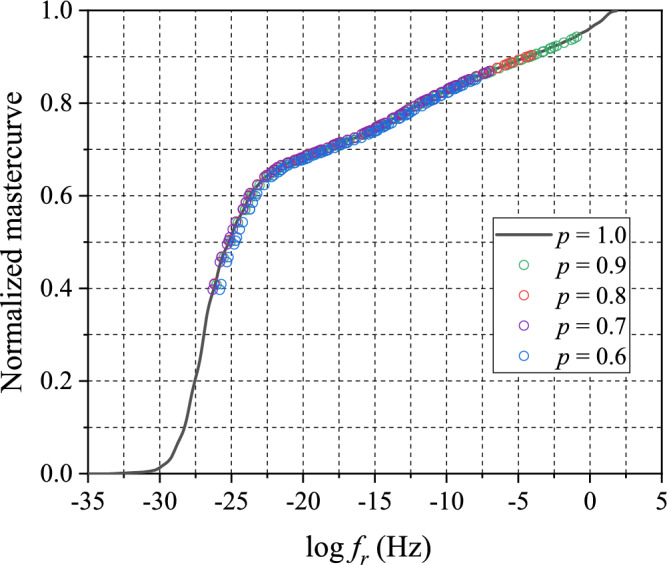
Results of TCS shifting using the master curve of fully-cured specimen as a reference.

The TCS shift factors were determined by shifting the master curves of partially-cured specimens to overlap with the master curve of fully-cured specimen $(p=1)$. The results of TCS shifting are shown in Fig. 11. The master curves show excellent overlaps indicating that there was no or negligible additional curing during the tests.

**Figure 12:**
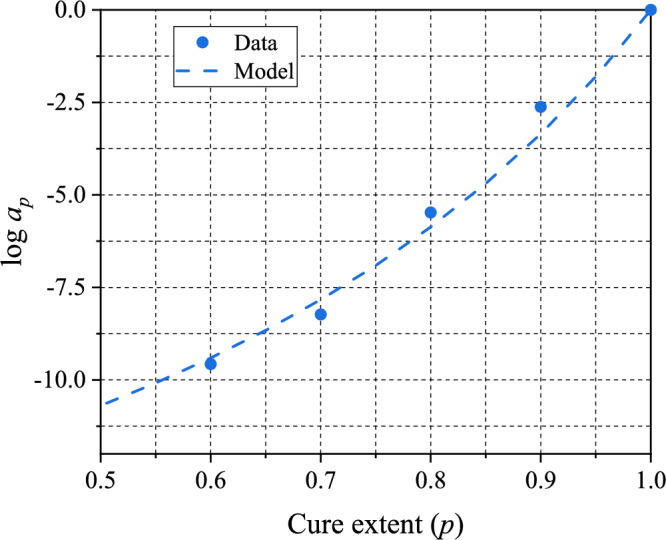
Time-cure superposition shift factors.

The corresponding TCS shift factors are shown in Fig. 12. The Williams-Landel-Ferry (WLF) function is one of the most widely used functions for shift factors [32]. Considering the behavior of the shift factors, a modified form of the WLF function is proposed to describe the TCS shift factors. The modified form is expressed as:

14 $$\log a_{p} \left( p \right) = \frac{{ - C_{1} \left( {T_{g\infty } - T_{g} \left( p \right)} \right)}}{{C_{2} + \left( {T_{g\infty } - T_{g} \left( p \right)} \right)}}$$

where ${T}_{{g}_{\infty }}$ is the glass transition temperature of fully-cured specimen; ${T}_{g}(p)$ is the glass transition temperature of partially-cured specimens; and ${C}_{1}$ and ${C}_{2}$ are material constants.

The constants were determined using the data in Fig. 12: they are ${C}_{1}=1.5036$ and ${C}_{2}=10.905$. The model prediction is also shown in Fig. 12. The model describes the TCS shift factors faithfully. The function is available in commercial FEA softwares, which is an added benefit in numerical implementation.

## Validity of TCS simplicity for EMC material

The time-cure superposition (TCS) process is based on the theoretical simplicity that cure-extent does not alter the relaxation mechanism, which implies that the TCS shift factors are cure-extend independent, i.e., ${e}_{i}(p)\approx {e}_{i}$. This theoretical foundation for the simplicity is solid, but it is important to confirm if the simplicity works ideally for the highly-filled EMC material tested in the study.

For the lowest cure-extent used in the study, $p=0.6$, the glass transition temperature, ${T}_{g}\left(0.6\right)$ is only around $60^\circ \mathrm{C}$, where the curing rate is extremely slow while the specimen exhibits very fast relaxation behavior. By taking advantage of the conditions of the partially-cured specimen at $p=0.6$, two additional frequency sweeping tests were conducted at $60 \text{ and } 70^\circ \mathrm{C}$ to produce a much larger section of the storage modulus master curve.

**Figure 13:**
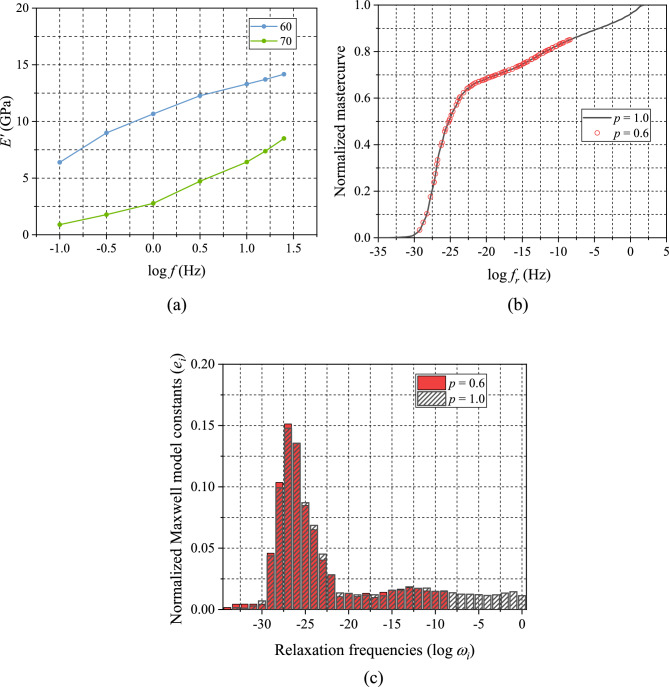
Analysis of partially-cured specimen at $p=0.6$: **a** Additional MFT test data; **b** shifting results with respect to $p=1.0$ after normalization; and **c** the normalized Maxwell model constants together with the constants obtained at $p=1$ (Fig. 6c).

The results of the additional testing are shown in Fig. 13a. Using this data as well as the data presented in Fig. 8a, a larger portion of storage modulus master curve at $p=0.6$ was constructed. The curve was normalized, and it was shifted using ${a}_{p}(0.6)$. The results is shown in Fig. 8b. The entire normalized master curve overlaps well with the normalized master curve of fully-cured EMC ($p=1$).

To verify the validity more quantitatively, the normalized Maxwell model constants determined from the normalized master curve of $p=0.6$ are compared with the constants of the fully-cured EMC that were determined in Sect. “MFT sweeping Tests of Fully and Partially-cured Specimens” (Fig. 6c). The results are plotted in Fig. 13c. The two sets of constants are nearly identical, which corroborates the validity of the TCS simplicity for the EMC tested in the study.

## Discussion: applicability of TTS to partially-cured specimens

In this study, the time–temperature superposition (TTS) shift factors, ${a}_{T}$, were assumed to be cure-extent independent, i.e., ${a}_{T}(T, p)={a}_{T}\left(T\right)$. This assumption is very reasonable under the TCS simplicity. Yet, it is instructive to investigate the assumption quantitatively.

**Figure 14:**
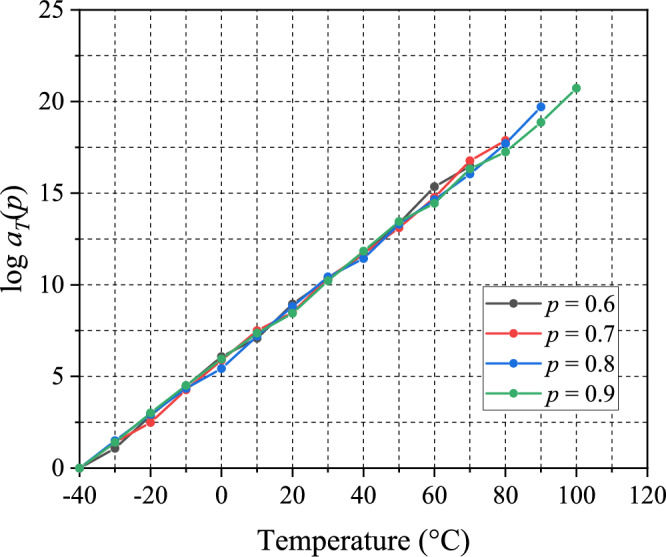
Time–temperature shift factors, ${a}_{T}$, obtained while constructing the master curves of the partially-cured specimens are plotted as a function of temperature.

The values of ${a}_{T}(T,p)$ were determined while constructing the sections of the master curves of the partially-cured specimens, as shown in Fig. 9. They are plotted as a function of temperature in Fig. 14. The results clearly show that the time–temperature shift factors, ${a}_{T}$, indeed have no or negligible cure dependency, which corroborates the validity of the approach used in the study.

## Conclusions

This paper proposed a novel method to measure the cure-extent dependent shift factors, ${a}_{p}(p)$, under *isocure* test conditions. The goal was achieved successfully by optimizing a test procedure while offering sufficient relaxation but producing no or negligible additional curing during testing.

The method was implemented using an epoxy-based molding compound. The cure kinetics behavior in the chemically-controlled domain was characterized first using the K-S autocatalytic model. Several partially-cured specimens were fabricated based on the model. The cure kinetics behavior was further characterized under the diffusion-controlled domain, and a new logistics function-based model was proposed to describe the curing behavior that accounted for both domains. The results were utilized to determine the maximum allowable test temperature, ${T}_{\mathrm{max}}$, below which no or negligible additional curing was expected during testing. Multi-frequency temperature sweeping (MFT) tests were conducted below ${T}_{\mathrm{max}}$ to produce portions of the storage master curves at four partially-cured states ($p=0.6, 0.7, 0.8, 0.9$). The curves were normalized by the corresponding equilibrium modulus and were subsequently shifted to determine the time-cure superposition (TCS) shift factors using the master curve of fully-cured specimen as a reference.

The results showed excellent overlaps over the entire curing range after shifting, corroborating that the proposed method eliminated the measurement uncertainties associated with the existing techniques.

The validity of TCS for the EMC material was examined using the additional testing data at the lowest cure-extent, ($p=0.6$). The analysis confirmed that the TCS fundamentals are suited ideally to the EMC material.

This study assumed that the time–temperature superposition (TTS) shift factors, ${a}_{T}$, were cure-extent independent. This assumption was investigated using the TTS shift factors obtained from the partially-cured specimens. As expected from the validity of TCS for the EMC material, the analysis clearly showed that the TTS shift factors, ${a}_{T}$, have negligible cure dependency, which corroborated the validity of the approach used in the study.

## Data Availability

The data that support the findings of this study are available on request.
